# Cell Origin and Culture History Determine Successful Integration of Neural Precursor Transplants into the Dentate Gyrus of the Adult Rat

**DOI:** 10.1371/journal.pone.0017072

**Published:** 2011-02-16

**Authors:** Xia Chen, Aviva M. Tolkovsky, Joe Herbert

**Affiliations:** Cambridge Centre for Brain Repair, University of Cambridge, Cambridge, United Kingdom; University of Cambridge, United Kingdom

## Abstract

The success of transplants of neural tissue into the adult dentate gyrus in generating mature neurons is highly variable. Here we address the roles of the origin of the tissue and its pre-implantation preparation, and show that both are critical. We transplanted neonatal cultured or primary rat cells from either the ventral subventricular zone (vSVZ) or the dentate gyrus (DG) into the adult rat DG. Only primary DG cells robustly generated DG neurons (80% NeuN and Prox1-positive cells at 6 weeks), substantially repaired the damaged DG, and formed glutamatergic projections to the target CA3 region. Cultured DG cells expanded for 7 days showed limited neuronal differentiation after transplantation (10% NeuN and Prox1-positive cells) whereas cultured or primary vSVZ cells failed to make any Prox1-positive DG granular neurons. We found that a specific population of postmitotic young neurons (triple doublecortin/NeuN/Prox1-positive) were particularly abundant in primary DG cells, but were markedly reduced in the cultured DG cells and were absent in the cultured and primary vSVZ cells. Labelling of primary DG cells with the mitotic marker BrdU suggested that postmitotic young neurons are the source of the transplanted mature neurons *in-vivo*. We conclude that both the origin and pre-transplantation history of donor cells are key factors that determine the outcome of transplantation. These findings may be of therapeutic interest for cell replacement therapy in treating the damaged hippocampus.

## Introduction

Neuronal progenitor cells (NPCs) in the dentate gyrus (DG) of hippocampus and the subventricular zone (SVZ) of the lateral ventricle continue to generate new neurons throughout adult life, a process known as adult neurogenesis [1], [2]. As well as being a source of NPCs, the DG is a region hospitable to the sustenance and maturation of new neurons [3]. Adult neurogenesis in the DG is impaired in several neurological diseases, resulting in neuronal cell loss [4], [5]. These lost neurons could possibly be replaced by transplanted NPCs [6].

The NPCs used in many transplantation studies are customarily expanded in mitogen-containing medium to increase cell number [7], [8]. Cultured NPCs provide a potentially unlimited source of cells for transplantation. Many studies have explored their ability to integrate into the host *in-vivo* [9], [10], [11], [12], [13]. Though some neuronal differentiation has been observed, this is generally limited and the prominent result has been differentiation into glial cells [12], [13], [14]. In contrast, transplantation of primary DG cells has resulted in substantial repair of the damage caused to the DG blade by the implantation procedure and this repair was not observed using cultured cells [15], [16]. The phenotype of the cells that gave rise to the repair following primary cell transplant has not been examined.

The origin of donor cells is a second factor that needs to be taken into account. [17], [18], [19], [20]. Compared to DG, SVZ contains a larger reservoir of NPCs and therefore represents an attractive donor source [21]. However, whether they represent an equivalent alternative source to site-specific DG tissue for repair of the DG remains to be determined. There are marked differences in the fate of the two sets of cells *in vivo*. The NPCs in the ventral SVZ (vSVZ) migrate to form inhibitory interneurons in the olfactory bulb, whereas those in the DG form excitatory glutamatergic neurons in the granular layer [3]. Accumulating evidence suggests that NPCs from different regions of the brain are intrinsically distinct [19], [22], [23]. For example, homotypic transplantation of SVZ NPCs has consistently generated olfactory bulb interneurons, whereas heterotypic transplantation into non-neurogenic regions such as striatum has not led to the generation of site-specific neurons [17], [19], [24]. Heterotypic transplantation into neurogenic regions such as DG may have a more favourable outcome, though this remains to be firmly established. [24].

In this study, we focus on addressing two questions: (i) given that NPCs are a mixture of cells at various stages of neuronal commitment, how does culture alter the composition of NPCs and how would this affect the outcomes of transplantation? (ii) are vSVZ NPCs in a heterotypic transplantation site capable of responding to the local environment to make DG-specific neurons? To address these questions, we transplanted four types of cells into the adult DG – cells from cultured vSVZ or cultured DG, and primary vSVZ or primary DG cells. We compared the outcomes of transplantation and demonstrate that both culture and origin of cells can affect the success of transplantation in terms of the phenotype of surviving cells, and the degree to which they can repair a damaged DG. By correlating the results of transplantation with the characteristics of donor cells from each of the four sources, we identified a specific population of cells that seem particularly capable of generating neurons and repairing the damaged DG.

## Results

### Transplantation of cultured vSVZ cells

**Figure 1:**
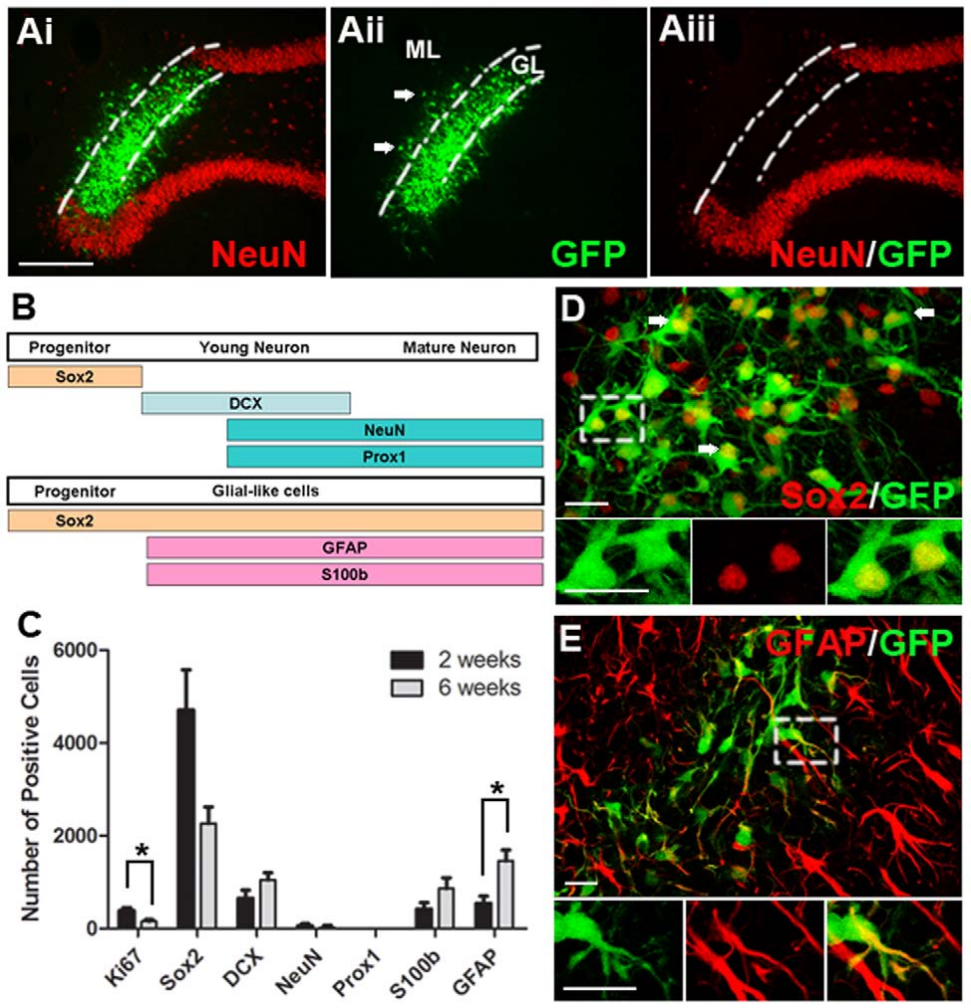
Transplanted cultured vSVZ cells fail to become DG granular neurons *in-vivo*. vSVZ cells from neonatal GFP rats were expanded for 7 days as neurospheres before transplantation into the DG of adult rats. (Ai-iii) Example of a GFP-positive vSVZ cell transplant in the hippocampus 6 weeks after grafting. Note the absence of NeuN staining in the granule cell layer (GL), indicating lack of replacement of damaged area by endogenous NPC pools. Some GFP+ vSVZ cells were not in the granule cell layer (GL) but were misplaced in the molecular layer (ML). (B) Diagram showing temporal pattern of expression of markers during differentiation into dentate gyrus (DG) granular neurons or into glial-like cells. (C) Total number of grafted cells per animal expressing different markers at 2 and 6 weeks (mean ± SEM, n = 4), *p<0.05. Note that many cells were positive for the progenitor cell marker Sox2, but no cell was positive for the DG granular neuronal marker Prox1. (D) Example of grafted Sox2+ cells 6 weeks after grafting. Panels below are enlargements of the boxed region. (E) Example of a grafted GFAP+ cell 6 weeks after grafting. Panels below are enlargements of the boxed region. Scale bar  = 100 µm for Ai-Aiii; scale bar  = 20 µm for D and E.

The fate of cultured vSVZ cells was investigated 2 and 6 weeks after transplantation. The suprapyramidal blade of the granular layer around the infusion site was damaged by the procedure and had lost the majority of granular cells expressing the neuronal marker NeuN (Neuronal Nuclei) (Figure 1A). Similar lesions were found in control experiments in which dead cells were injected and also in the later experiments irrespective of which cell types were injected, indicating that the damage was due to the infusion regime [15]. Staining for GFAP showed heightened glial activation around this region (as expected [25]), irrespective of whether and which cells had been injected. Damage after the control infusions persisted up to 6 weeks post-injection, showing that the endogenous NPCs could not replace the damaged cells within this time period.

At 2 and 6 weeks post-transplantation, 7476±1470 cells and 4846±1904 cells were present in the adult DG, a survival rate of 15.0±2.9% and 9.7±3.8%. Many transplanted cells expressed the progenitor cell marker Sox2 (sex determining region Y-box 2; mean 63% at 2 weeks), which decreased between 2 to 6 weeks (p = 0.06, Figure 1C,D). In contrast, there was a significant increase in cells expressing the glial cell-related marker GFAP (glial fibrillary acid protein; p<0.05, Figure 1C,E), indicating some differentiation into glial cells. Although some cells expressed the immature neuronal marker DCX (double-cortin) (9% at 2 weeks, Figure 1C), very few cells expressed the more mature neuronal marker NeuN at both 2 and 6 weeks (Figure 1A,C), indicating very little maturation had occurred. No cells expressed the DG granular neuronal marker Prox1 (Prospero homeobox protein 1) (Figure 1C) [26], suggesting that cultured vSVZ cells were unable to generate DG granular neurons. Only a few cells expressed the proliferation marker Ki67 (5% at 2 weeks), which decreased further between 2 and 6 weeks (p<0.05, Figure 1C).

The migration of implanted cells was also investigated. DCX+ cells were not restricted to the granular cell layer, where endogenous cell bodies are located. Some had migrated ectopically into the molecular layer (Figure 1A), where the dendrites of the granular cells reside, suggesting that the cells were unable to respond to local signals regarding positioning. Staining of GFP+ (green-fluorescent protein) axons in the CA3 region was not observed, thus indicating lack of projections to the target region of granular DG neurons. Together, these data suggest that cultured vSVZ were unable to produce site-specific DG granular neurons *in-vivo*. We therefore performed homotypic transplantation of cultured DG cells to test whether they could give rise to mature DG granular neurons.

### Transplantation of cultured DG cells

**Figure 2:**
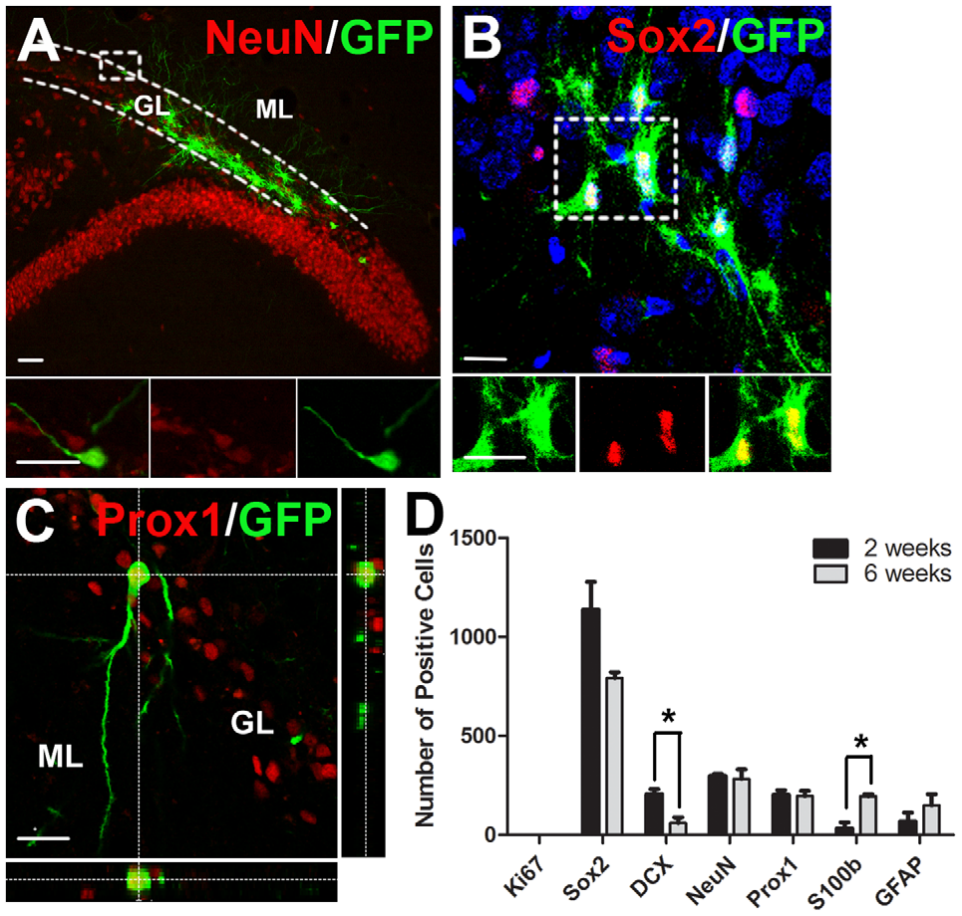
A small minority of transplanted cultured DG cells become DG granular neurons *in-vivo*. The DG was dissected out of newborn GFP+ rats and expanded for 7 days as neurospheres. The cultures were dispersed and transplanted into the DG of adult rats. (A) Examples of GFP+ cell transplants 6 weeks after grafting. Some grafted cells are expressing NeuN and they are all found in granular layer (GL). (B) Some grafted cells in the subgranular zone expressing the progenitor cell marker Sox2. (C) Image of grafted cell expressing the DG granular cell marker Prox1. Note that the Prox1+ cell has extended dendrites into molecular layer and is morphologically similar to the endogenous neurons. (D) Total number of grafted cells per animal expressing the different markers at 2 and 6 weeks (mean ± SEM, n = 5), *p<0.05. Scale bar  = 20 µm for A–C.

At 2 and 6 weeks post-transplant, 2131±310 cells and 1746±473 cells remained in the adult DG respectively, a survival rate of 5.3±1.8% and 4.3±0.6%. Similar to vSVZ cells, many transplanted cultured DG cells expressed the neuronal progenitor marker Sox2 (53% at 2 weeks), which decreased between 2 and 6 weeks (p = 0.07, Figure 2B,D). Alongside this decrease, there was a significant increase in S100β+ (S100 calcium binding protein B) cells (p<0.05, Figure 2D), indicating some differentiation into glial cells. Unlike cultured vSVZ, some transplanted cultured DG cells expressed Prox1 (10%), which remained stable at 6 weeks (Figure 2C,D). A similar percentage (14%) also expressed NeuN+ up to 6 weeks (Figure 2A,D). In contrast, DCX+ cells decreased significantly between 2 and 6 weeks (p<0.05, Figure 2D). We found that the majority of the Prox1+ (82.0±2.3%) and NeuN+ (71.8±1.9%) cells were positive for DCX+ at 2 weeks, suggesting that they were young neurons. The reduction in DCX+ cells and the maintenance of Prox1+/NeuN+ cells up to 6 weeks indicate the neuronal maturation of DCX+/Prox1+/NeuN+ cells to DCX-negative Prox1+/NeuN+ cells. These Prox1+ NeuN+ cells were found mostly in the intact region of the DG, in keeping with previous data [13]. No proliferating Ki67+ cells were observed (Figure 2D).

Interestingly, unlike implanted vSVZ cells, the position of the transplanted NeuN+/Prox1+ cultured DG cells was confined to the granular cell layer (Figure 2A), and the neurons were morphologically similar to endogenous neurons (Figure 2C). Dendrites extending to the molecular layer could be observed (Figure 2C). However, there was little evidence for GFP+ axons in the target CA3 region, though there were very few GFP+ neurons so the endings may have been missed due to their vast dilution within the CA3 layer. Although, unlike cultured vSVZ cells, cultured DG cells were able to generate Prox1+ DG granular neurons, the extent of survival and generation of mature neurons was quite low. We therefore transplanted primary vSVZ or primary DG cells to examine whether survival and/or neuronal differentiation could be improved.

### Transplantation of primary vSVZ cells

At 2 and 6 weeks post-transplant, 13486±1918 cells and 7035±1287 cells were present in the adult DG respectively, a survival rate of 27.0±3.8% and 14.1±2.2%. Compared to cultured vSVZ cells, primary vSVZ showed a slightly higher survival rate at 2 weeks (p = 0.07), but this difference became much narrower at 6 weeks, indicating that many cells continued to die.

**Figure 3:**
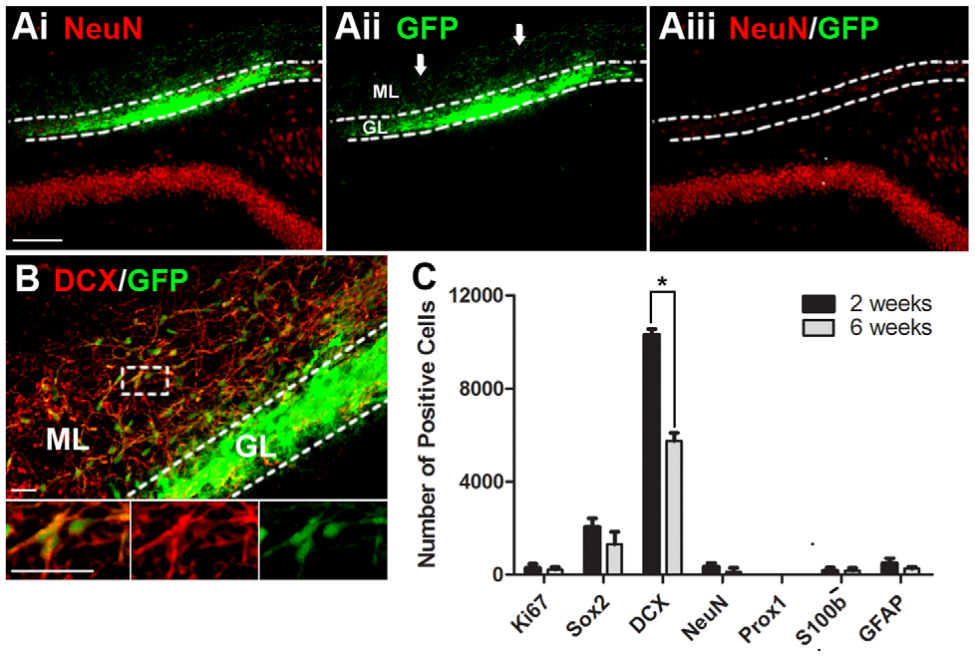
Transplanted primary vSVZ cells fail to become DG granular neurons *in-vivo*. vSVZ cells were isolated from neonatal GFP rats and transplanted immediately after isolation. (Ai–Aiii) Example of a GFP-positive vSVZ cell transplant in the hippocampus 6 weeks after grafting. Note that the transplanted cells did not repair the damage in the granular layer (GL). Note also that many of these DCX+ cells have migrated beyond the granular layer into the molecular layer (ML). (B) Example of grafted cell expressing the immature neuronal marker DCX. (C) Total number grafted cells per animal expressing different markers at 2 and 6 weeks (mean ± SEM, n = 4), *p<0.05. Note that few cells expressed NeuN and no cells expressed Prox1. Scale bar  = 100 µm for A; scale bar  = 50 µm for B.

Many primary vSVZ cells expressed the immature cell marker DCX (76% at 2 weeks), which decreased significantly between 2 and 6 weeks (p<0.05, Figure 3B,C). However, this did not lead to an increase in NeuN+ cells and none of the cells expressed the DG granular neuronal maker Prox1 (Figure 3C). To examine whether these DCX+ cells were making GABAergic interneurons instead, which would be their fate in situ, we stained the cells for the expression of glutamic acid decarboxylase 67 (GAD67). We found that only a few cells expressed GAD67 (3±1.2%) at 6 weeks, suggesting that these DCX+ young neurons did not mature further but rather had died. Some cells expressed Sox2 at 2 weeks (15%), which decreased slightly but not significantly at 6 weeks (p = 0.3, Figure 3C). However this did not lead to an increase in the numbers of cells expressing the glial cell-related markers GFAP and S100β at 6 weeks (Figure 3C), indicating differentiation into glial cells was negligible. Very few cells expressed Ki67 at 6 weeks, indicating that there was little proliferation.

Similar to cultured vSVZ cells, DCX+ cells from primary vSVZ transplants migrated beyond the granular cell layer and into the molecular layer (Figure 3A), suggesting they were unable to respond to local cues governing positioning. No obvious staining of GFP+ processes was observed in the CA3 region. Also similar to cultured vSVZ cells, primary vSVZ cells were also unable to make site-specific Prox1+ DG granular neurons and the cells continued to die between 2 and 6 weeks. We next transplanted primary DG cells to test whether they would show a different pattern of neuronal formation and survival.

### Transplantation of primary DG cells

At 2 and 6 weeks post-transplant, 13184±1113 and 12057±1780 cells were found to be present in the adult DG respectively, a survival rate of 26.4±2.2% and 24.1±3.6%. This was a significantly higher survival rate than that of cultured DG cells (p<0.001) or cultured vSVZ cells (p<0.05) at 2 and 6 weeks, being rather similar to that found for primary vSVZ cells at 2 weeks but better than vSVZ survival at 6 weeks.

**Figure 4:**
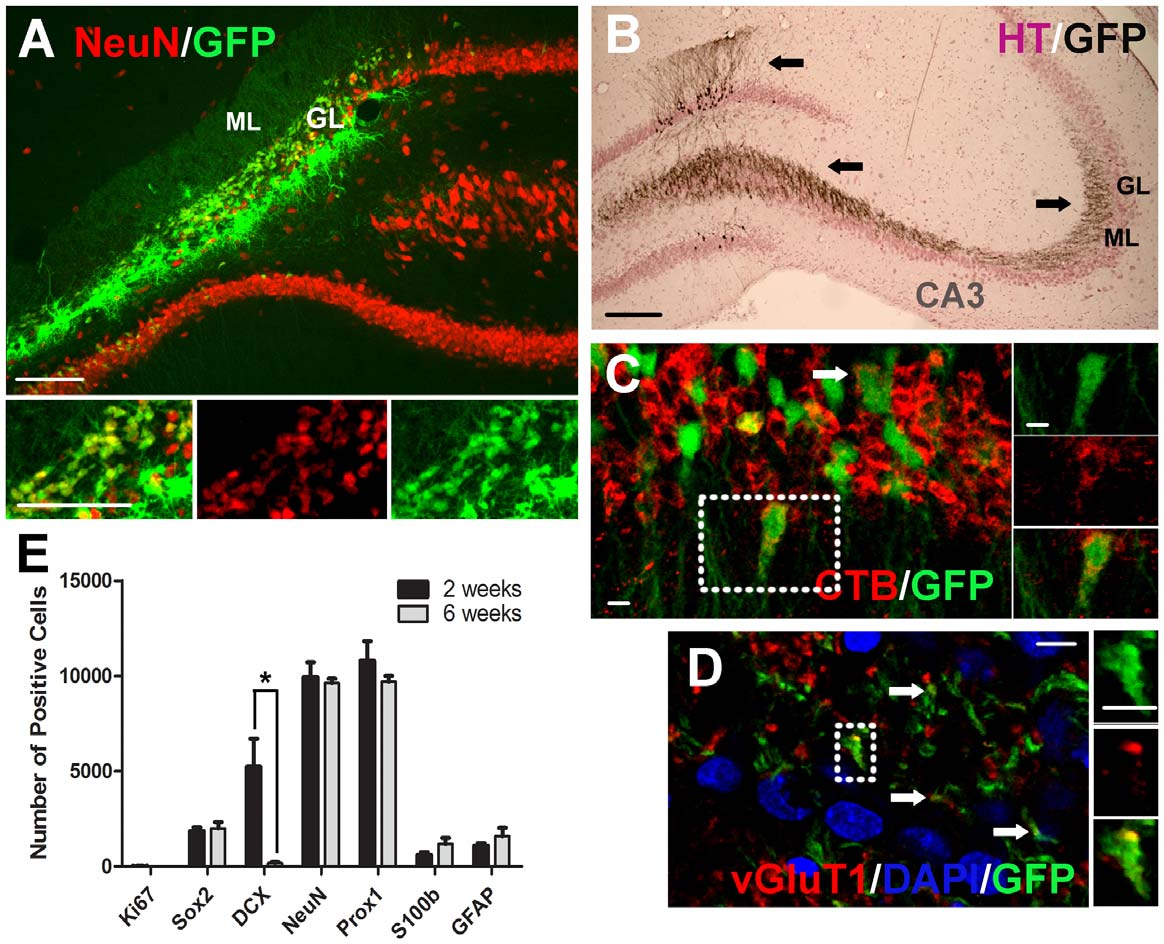
Most of the transplanted primary DG cells become DG granular neurons *in-vivo*. DG cells were isolated from neonatal GFP rats and transplanted immediately after isolation. (A) Example of a GFP+ cell transplant in the hippocampus 6 weeks after grafting. Note that the grafted cells have substantially replaced missing NeuN+ cells in the granular layer. (B) Immunostaining of GFP in grafted cells detected with DAB (black). The section was counterstained with haematoxylin (HT) (purple) to detect cell nuclei. Note the substantial projection of transplanted neurons to the target CA3 region and intense ramification therein, as well as well developed dendritic projections into the molecular layer. (C) Retrograde transport of tracer cholera toxin B (CTB) injected into CA3 confirming this projection. (D) Image showing the expression of the pre-synaptic vesicular glutamate transporter 1 (vGluT1) on the axon terminals of grafted cells. (E) Total number of grafted cells per animal expressing different markers at 2 and 6 weeks (mean ± SEM, n = 4–5), *p<0.05. Note that the majority of cells are positive for NeuN and Prox1. Scale bar  = 100 µm for A–B; scale bar  = 10 µm for C–D.

Unlike cultured DG cells, most primary DG cells expressed NeuN (75%, Figure 4A,E) and Prox1 (89%, Figure 4E) at 2 weeks, and this expression persisted at 6 weeks. Similar to cultured DG cells, DCX+ (40% at 2 weeks) decreased significantly by 6 weeks (p<0.05, Figure 4E). We found that about half of the NeuN+ (56.2±3.1%) and Prox1+ (48.5±2.5%) cells were also positive for DCX+ cells at 2 weeks, suggesting they were young neurons. The reduction in DCX+ cells and the maintenance of NeuN+ Prox1+ cells at 6 weeks indicated neuronal maturation of these cells. Cells expressing the progenitor cell marker Sox2 (14% at 2 weeks), or the glial cell-related markers GFAP (8%) and S100β (5%) appeared to be stable populations as their numbers did not change up to 6 weeks (Figure 4E), indicating limited differentiation. No mitotic Ki67+ cells at either 2 or 6 weeks were observed (Figure 4E).

The majority of transplanted cells remained within the damaged site of the DG but some migrated ventrally into the intact blade. At both time points, cells were located mostly within the granular cell layer (Figure 4A,B). Notably, the newly generated DCX-negative, NeuN+/Prox1+ granular neurons in the damaged site had extensively repopulated and regenerated the damaged part of DG (Figure 4A). Granular neurons formed by these transplanted cells were phenotypically similar to endogenous cells. They extended dendritic branches into the molecular layer and numerous GFP+ axons could be observed in the target CA3 region at both 2 weeks and 6 weeks (Figure 4B). The origin of these projections was confirmed by the presence of the tracer cholera toxin B in the cells after it had been retrogradely transported from its injection site in CA3 (Figure 4C). Also similar to endogenous cells, the granular neurons formed by the transplanted cells were glutamatergic, expressing the pre-synaptic vesicular glutamate transporter 1 (vGluT1, Figure 4D) on their axon terminals in CA3. Hence, these primary transplants were morphologically no different from the endogenous DG neurons.

### Phenotypes of donor cells

**Figure 5:**
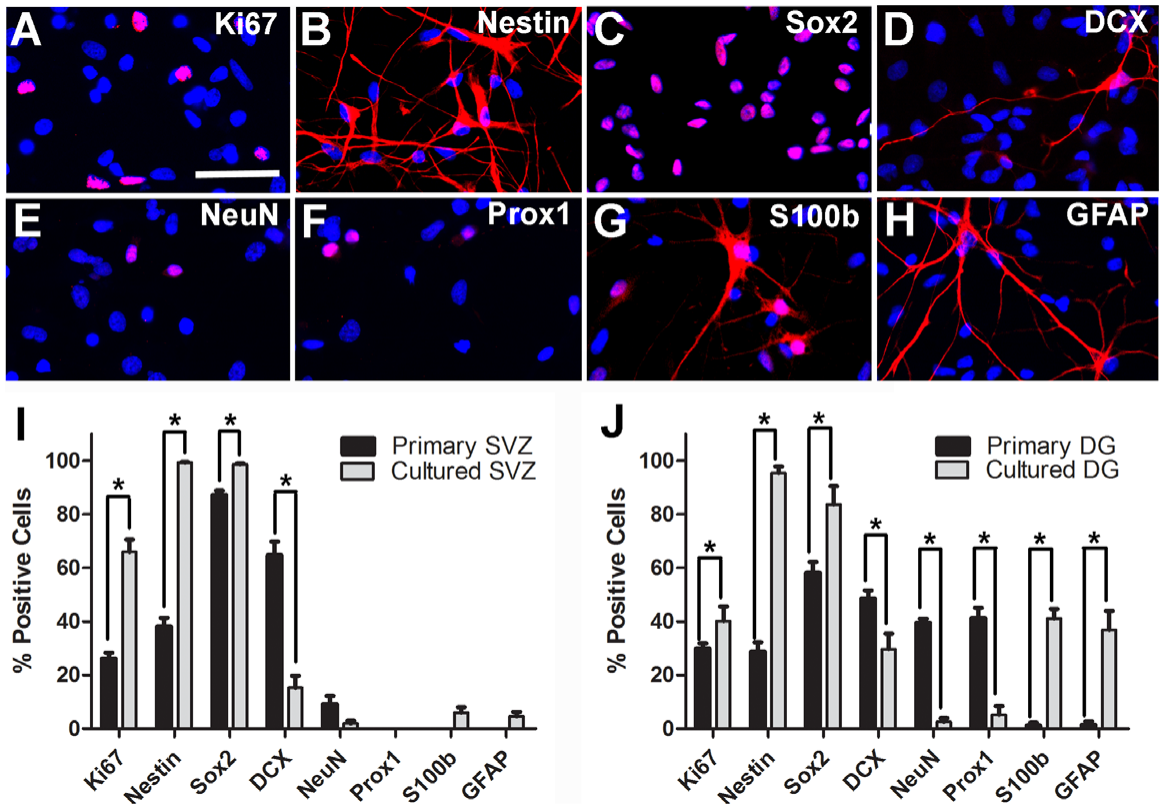
Primary DG donor cells are the only cell type that contains a subpopulation of NeuN/Prox1+ cells before transplantation. Examples of cultured DG cells stained for (A) the proliferation maker Ki67, (B–C) the progenitor cell markers Nestin and Sox2, (D–F) the neuronal cell markers DCX, NeuN and Prox1 and (G–H) glial-related markers S100β and GFAP. (I–J) Percentages of cultured or primary DG and vSVZ cells expressing different markers (mean ± SEM, n = 3), *p<0.05. Scale bar  = 50 µm.

To further investigate the outcomes following transplantation of different tissues, we compared the characteristics of the donor cells from the four origins. Compared to primary cells, cultured cells contained more mitotic cells expressing Ki67 (p<0.05 for vSVZ, p = 0.13 for DG, Figure 5I,J). Cultured cells also contained more progenitor cells expressing Nestin (p<0.05 for vSVZ and DG) and Sox2+ (p<0.05 for vSVZ and DG) (Figure 5I,J). In contrast, cultured cells contained fewer neuronal cells expressing DCX (p<0.05 for vSVZ and DG), NeuN (p = 0.08 for vSVZ and p<0.05 for DG) and Prox1 (p<0.05 for DG) (Figure 5I,J). These data suggest that during culture, the populations of Nestin+ and Sox2+ progenitor cells were expanded whereas those expressing DCX, NeuN or Prox1 were either diluted or not sustained.

Among the four cell types, the populations of DCX+, NeuN+, and Prox1+ cells were particularly abundant in primary DG cells (49% DCX+, 40% NeuN+ and 41% Prox1+, Figure 5J). These populations of cells were significantly reduced in cultured DG cells (30% DCX+ p<0.05, 3% NeuN+ p<0.05 and 5% Prox1+ p<0.05, Figure 5J) and they were absent in the cultured or primary vSVZ cells (Figure 5I). Furthermore, we found that the populations of DCX+, NeuN+ and Prox1+ cells largely overlapped, as most DCX+ cells in primary DG were also positive for NeuN (65.6±2.3%) and Prox1 (78.0±1.3%). Likewise, almost all of the NeuN+ (91.5±5.4%) and Prox1+ cells (97.6±2.4%) were positive for DCX, suggesting these cells represented immature neurons.

Only primary DG cells contained a high level of DCX+, NeuN+ or Prox1+ cells before transplantation. We therefore hypothesized that this population of DCX+, NeuN+ and Prox1+ cells were the ones that robustly formed DG granular neurons after transplantation. To test this hypothesis, we labelled primary DG cells with BrdU before transplantation to determine whether cells re-populating the DG were post-mitotic at the time they were transplanted.

### BrdU labelling of primary DG cells

**Figure 6:**
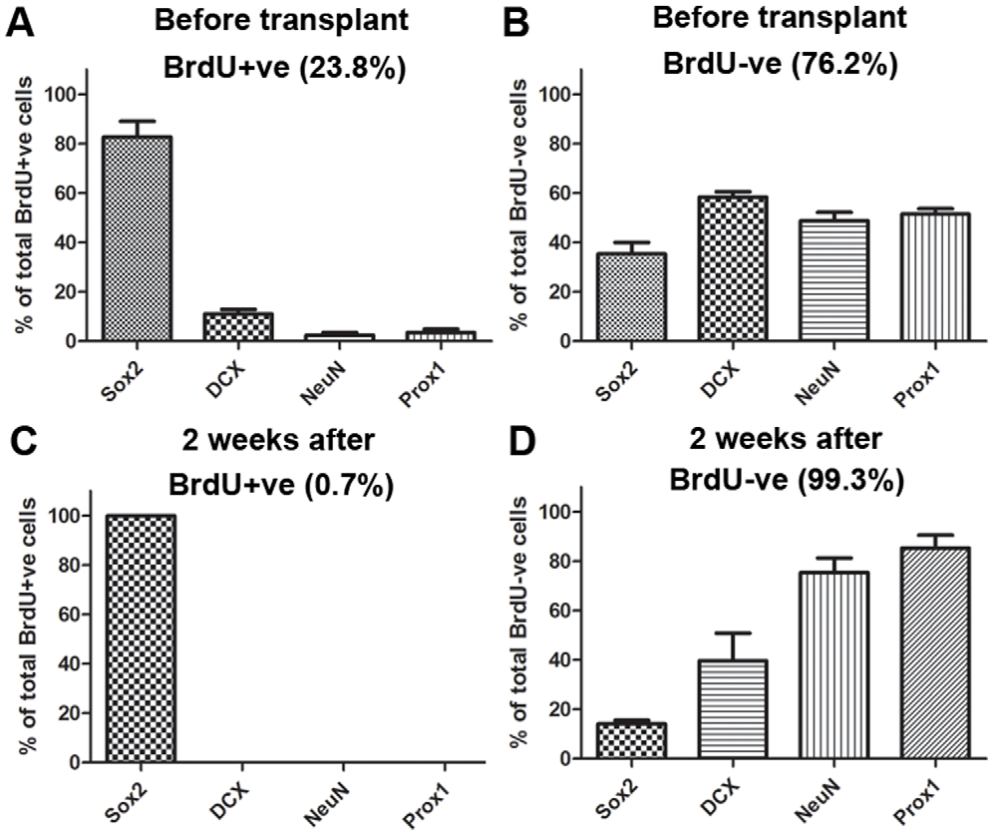
Most of the NeuN+/Prox1+ primary DG cells are postmitotic prior to transplantation. Pups were injected with BrdU 5–7 hours prior to sacrifice and harvesting of cells. (A) Percentages of BrdU+ cells and (B) BrdU -ve cells expressing Sox2, DCX, NeuN and Prox1 before transplantation. (C) Percentages of BrdU+ cells and (D) BrdU –ve cells expressing these markers at 2 weeks after transplantation. The total percentages of BrdU+ and BrdU –ve cells are shown in brackets. Note that while most of the Sox2+ cells were labelled with BrdU, and these survived transplantation, very few DCX+/NeuN+/Prox1+ cells were labelled before transplantation, and no labelled cells expressing these markers were detected 2 weeks after transplantation.

A single injection of BrdU was given to donor (neo-natal) rats 5–7 hours before the tissue was collected. This BrdU injection would be expected to label mainly dividing cells but fewer DCX+, NeuN+ and Prox1+ cells, which would be mostly post-mitotic. BrdU labelled 23.8±3.5% of the primary DG cells. Among these BrdU+ cells, most were progenitor cells expressing Sox2 (84% of BrdU+ cells) whereas only a few expressed DCX (11%), NeuN (2%) or Prox1 (3%) (Figure 6A). In contrast, the BrdU-negative population contained much higher percentages of cells expressing DCX (58%), NeuN (50%) and Prox1 (51%) (Figure 6B). At 2 weeks post-transplantation, the majority of the surviving cells were BrdU-negative (99.3%). Among these, the majority were positive for NeuN (75%) and Prox1 (85%) (Figure 6D). Fewer cells were positive for DCX (40%, Figure 6D), suggesting that some neuronal precursor cells had matured. The few remaining BrdU+ cells (0.7%) were all positive for Sox2 (100%) and negative for NeuN (0%) or Prox1 (0%) (Figure 6C). Together, these data suggest that the DG granular neurons generated by transplanted primary DG cells were likely to come from the population of non-mitotic DCX+, NeuN+ and Prox1+ cells in the transplants.

## Discussion

This paper shows that there are two distinct determinants of the survival and maturation of transplants into the adult dentate gyrus from two neurogenic regions of the brain. Primary DG cells were the most robust, giving rise to the greatest number of surviving cells, the most advanced differentiated phenotype, and the only cells able to re-populate a damaged region of the dentate gyrus. Comparison of the phenotypes of donor cells identified a population of young neurons expressing DCX, NeuN and Prox1, which were particularly abundant in the primary DG cells, but were greatly reduced in cultured DG cells and absent in vSVZ cells of either source. BrdU labelling showed that these cells were mostly post-mitotic at the time of transplantation. The inference is that young neurons expressing DCX, NeuN and Prox1 represented the population of cells that enabled primary DG transplants to form DG neurons so robustly after transplantation. This idea is supported by the finding of very low numbers of these cells in the cultured DG population, together with the very low percentage of Prox1 cells (which nevertheless persist) that appeared after transplantation. This finding, together with an examination of the phenotype of the transplanted cells, suggests that the culture conditions used prevented the appearance or persistence of the young neuron phenotype in primary DG cells, and furthermore that the vSVZ may not contain, or is unable to give rise to, this population.

The survival of cells was generally greater for primary cells than cultured cells from either region. Cultured cells exposed to 20% oxygen *in-vitro* might experience higher cell death than primary cells when transplanted back to the 3% oxygen environment in the brain [27]. The low survival of cultured cells in our study broadly agrees with earlier reports, in which the survival rate of transplanted cultured cells has ranged from less than 1% to around 15% in different systems [12], [13], [14]. Among all four cell types in our study, cultured DG cells had the lowest cell survival. Comparison of donor cells showed that cultured DG cells contained a particularly high number of S100β+ GFAP+ cells (∼40%) compared to vSVZ cells (<10%). These cells often displayed extensive processes *in-vitro* and might therefore be particularly susceptible to damage during mechanical dissociation, especially given that the DG neurospheres had a tighter structure and were thus more difficult to dissociate compared to vSVZ neurospheres. Consistent with this idea, many more of these cells were found to be lost from cultured DG than from vSVZ after transplantation, despite there being almost 4-fold fewer of these cells among the vSVZ donors. Compared to primary DG cells, the survival of primary vSVZ cells continued to decrease at 6 weeks, suggesting continuing mismatch of these cells within the heterotypic DG site.

Compared to primary DG cells, cultured DG cells showed limited neuronal differentiation following transplantation. This finding agrees with previous studies using cultured neuronal progenitor cells, where the percentages of β-III tubulin+ or NeuN+ cells varied from almost zero to around 30% overall [12], [13], [14]. Also consistent with other studies, neuronal differentiation of the cultured DG transplant occurred mainly in the intact region of the DG, suggesting that this may be a more supportive environment [13]. However, since primary DG transplants were able to repopulate the damaged layer, this area seemed to sustain maturation of young neurons, but was less hospitable to the initial stage of neuronal differentiation. Nonetheless, neither extensive neuronal differentiation of the cultured cells nor their ability to repair the damaged blade was observed. In almost all of the previous studies, differentiation into glial cells was the dominant event [12], [13], [14], [28]. Thus, the striking contrast in the number of surviving and maturing neurons between primary DG and cultured DG cells observed in this study strongly suggests that primary DG cell possess a distinct quality which was largely absent from cultured DG cells. The remarkable repair of the damaged blade of the dentate gyrus by the primary DG cells also indicates that the adult host environment (including glial activation) is not the limiting factor constraining this repair.

We showed that the DCX+, NeuN+ and Prox1+ postmitotic young neurons in the primary DG cells were likely to be the ones that were integrated into the host. The advantages of using committed young neurons rather than undifferentiated progenitor cells for successful cell replacement has also been described for dopamine neurons transplanted into the striatum [29], [30], [31]. Recent evidence supports the notion that these markers may represent features important for neuronal differentiation and maturation. The neuronal specific nuclear protein NeuN is now known to be a new member of the Fox1 gene family of splicing factors, which suggests it may have an important role in carrying out neuronal specific splicing in the regulation of neuronal maturation [32]. The homeobox gene Prox1 is expressed in the DG during development as well as in the adult [26], [33] and has been shown to have an important role in the maturation of granular cells in the DG [34]. It remains of interest to determine whether this population of DCX+, NeuN+ and Prox1+ cells are capable of generating other site-specific neurons if they are transplanted to different regions of brain. It was notable that the cells that were left in the tract through the cortex made by insertion of the cannula did not generate cortical neurons, which suggests this might not be the case, although the environment within the tract could have been altered by other factors, such as inflammation. Direct evidence testing this possibility will be required.

The critical window for survival of DG primary transplants appears to be during the first 2 weeks, since survival rates remained very much the same at 6 weeks. However, neuronal maturation continued to occur beyond the first 2 weeks, as suggested by the decrease in DCX+ cells and a shift from the less mature DCX+NeuN+Prox1 triple positive cells to the more mature DCX-negative, NeuN+, Prox1+ double positive cells. The new neurons formed by the transplanted primary (as well as cultured) DG cells resembled that of endogenous cells morphologically, suggesting that they were able to respond to cues from the local environment that govern both maturation and positioning within the dentate gyrus. However, it remains to be determined whether these cells are electrophysiologically active and whether repair at the morphological level has functional significance.

Although transplanted cultured and primary vSVZ cells contained DCX+ cells, the percentages of NeuN+ cells were low and no cells expressed Prox1 at 6 weeks. This was not likely to be due to insufficient time for differentiation since endogenous DG NPCs acquire NeuN expression 2 weeks, and Prox1 expression 1 week, after the expression of DCX [3], [34]. It appears therefore that vSVZ cells were incapable of maturing into DG specific neurons. Some cells expressed GAD67, which further suggested that vSVZ cells might be restricted to making cells similar to those they generate *in vivo*.

Increasing evidence suggests that NPCs in different regions of the brain are not homogeneous and that they are specified by both spatial and temporal attributes [17], [18], [19], [20], [22], [35], [36], [37]. Transplantation of primary SVZ cells, for example, resulted in extensive neuronal differentiation only in the olfactory bulb, but not in heterotypic regions of cortex and striatum [17]. In the cerebellum, although transplanted primary SVZ cells can acquire certain site-specific markers, a complete switch to cerebellar-specific neurons was not observed [19]. This regional specificity of NPCs occurs even in different regions of SVZ, along both anterior-posterior and dorsal-ventral axes [22], [38], [39]. For example, SVZ NPCs from the ventral region (the region sampled in these experiments) are known to make GABAergic interneurons, while the dorsal region was shown to make glutamatergic interneurons [38]. In preliminary experiments, we tested the capacity of dorsal SVZ to integrate into the DG but there was no discernible difference compared to vSVZ and no glutamatergic neurons developed.

Although we present suggestive data that the cell populations of DG and vSVZ are different, and that this difference may be directly related to the ability of these cells to survive and repopulate the adult DG, the exact processes that occur during culture which, by reducing the number of DCX+, NeuN+ and Prox1+ cells, impairs their survival, maturation and ability to re-populate a damaged DG remain to be explained. The determinants that confer integration of the DCX+, NeuN+ and Prox1+ cells into the DG remain to be identified. Ongoing studies in progress will test whether it is possible to convert cultured DG into appropriate precursors by enhancing the presence of NeuN+ and Prox1+ cells or by altering the environment of the host. We conclude that both the origin and subsequent treatment of transplanted donor cells contribute crucially to the fate of post-natal implants within the DG, although other factors such as the age of the donors might also play a contributing role. Importantly, we have defined a specific population of cells that undergoes robust integration into the host DG after transplantation.

## Materials and Methods

### Preparation of primary and cultured cells

Cell suspensions of either primary or cultured DG or vSVZ cells from postnatal rats were transplanted into the DG of adult rats. Donors were postnatal rats (P2-P4) expressing green fluorescent protein (GFP) ubiquitously under the control of the β–actin promoter, which allowed the identification of all transplanted cells. The brain was removed and placed in cold HBSS. Under a light microscope, the hippocampus was dissected out and the DG was separated out along the hippocampal fissure. For vSVZ, a thin layer of tissue was dissected out along the lower half of the lateral wall of the lateral ventricle rostral to the hippocampus. The DG and vSVZ tissues were then incubated in Accutase solution (Sigma) at 37°C for 10 min, followed by mechanical trituration to obtain single cell suspensions. Cell density was determined by haemocytometric counting. Viability of the cells was 78.0±5.4% as determined by trypan blue staining. In the experiments that involved the labelling of primary cells with the mitotic marker bromodeoxyuridine (BrdU), a single injection of BrdU (200 mg/kg in saline) was given IP 5–7 hours before the animals were euthanized. For one host adult rat, the brain from one pup was used as donor.

For neurosphere culture, brains from several pups were pooled together. Dissociated primary DG and vSVZ cells were seeded into non-coated flasks at a density of 100,000 viable cells per ml in medium containing Neurobasal A (Gibco), L-glutamine (1%, Invitrogen), B-27 supplement (2%, Gibco), human fibroblast growth factor -2 (FGF-2, 20 ng/ml, R&D Systems), heparin (5 µg/ml), human epidermal growth factor (EGF, 20 ng/ml, Sigma) and a penicillin-streptomycin-amphotericin mixture (1%, Gibco). Fresh (50% volume) growth medium was added on day 3 and the cells were maintained at 37°C in a humidified 5% CO_2_/95% air atmosphere for 7 days.

To examine the phenotypes of cells before transplantation, dissociated primary or cultured cells were plated onto glass coverslips precoated with laminin (10 µg/ml). To dissociate neurospheres, cells were treated with Accutase at 37°C for 10 min, followed by mechanical dissociation. Dissociated cells were plated at a density of 50,000 cells per coverslip in plating medium containing Neurobasal A, L-glutamine (1%), B27 (2%), fetal calf serum (FCS, 1%) and PSF (1%). Cells were then fixed 4 hours later in 0.1 M phosphate buffered saline (PBS) containing 4% paraformaldehyde (PFA) for 10 min followed by washing with 0.1 M PBS.

### Transplantation

All procedures were approved by the Cambridge local ethical committee and carried out under Home Office (UK) licence guidelines. Adult Sprague-Dawley (SD) male rats weighing 250–300 g were used as transplant recipients. Four to five animals were transplanted with each cell type. As controls, three SD rats received infusions containing dead cells of each cell type that had been through 6 freezing-thaw cycles. The recipient rats were anesthetized with isoflurane and placed onto a stereotactic apparatus (Kopf) with the mouth bar 3.5 mm below zero. Unilateral injections of 50,000 cells in 1 µl of Dulbecco's Modified Eagle's Medium was made into the DG with a SGE syringe (gauge 23, outer diameter 0.63 mm and inner diameter 0.37 mm) using the following coordinates: 3.6 mm posterior to bregma, 1.4 mm from the midline, and 4.0 mm below the dural surface. A smaller needle (gauge 26) was used initially, but it was found to be blocked by cells. One µl was injected over a course of 2 min and the needle was left for a further 2 min before being slowly withdrawn. After a survival time of 2 or 6 weeks, recipients were perfused with 4% PFA and brains were further fixed in 4% PFA overnight before being transferred to 30% sucrose. Coronal sections (25 µm) were cut using a freezing microtome starting from 1.5 mm rostral to the transplantation site to 1.6 mm posterior to it. Every 6^th^ section was checked for the presence of transplanted GFP positive cells by fluorescence microscopy. For tracing experiments, cholera toxin B (10 µg/ul, 1 µl, Invitrogen) was injected into the CA3 region at a rate of 0.2 µl per min using a micro-infusion pump three days before sacrifice.

### Immunostaining

Cells or tissues were stained using the following primary antibodies: anti-GFAP (9205), anti-GFP (A11122), anti-Prox1 (P0089, all from Sigma), anti-NeuN (MAB377, Chemicon), anti-Ki67 (VP-RM04, Vector), anti-BrdU (OBT0030, Accurate chemical) and anti-DCX (sc8066), anti-Sox2 (sc17320, both from Santa Cruz) were all used at 1∶500. Anti-S100β (ab52642), anti-GFP-FITC (ab6662, both from Abcam) and anti-vGluT1 (ab5905, Millipore) were used at 1∶300, 1∶500 and 1∶1000 respectively. DAPI (D9542, Sigma) was used to identify nuclei at 10 µg/ml. The secondary antibodies (donkey anti-rabbit Alexa 488/568/680, donkey anti-mouse Alexa 555/680, donkey anti-goat Alexa 568/647, donkey anti-rat Alexa 594 and donkey anti-guinea pig 568) were all from Invitrogen and were all used at 1∶500 dilution. As a control, cells or tissues were incubated with secondary antibodies only and no non-specific binding was observed. For DAB staining, tissues sections were incubated with biotinylated secondary rabbit IgG antibody used at 1∶000 and were visualized with an ABC kit (both from Vector).

### Quantification

To characterise cells *in-vitro* before transplantation, three coverslips each from three separate experiments were stained for each marker. On each coverslip, 5–7 images were acquired 1 mm apart using a fluorescence microscope (Leica) with ×40 objective. Images were opened in LAS AF Lite software and the number of marker positive cells was counted. Co-localization was determined by overlapping different channels and merging images. The percentage of positive cells out of the total GFP+ cells counted is presented for each marker. Because one cell may have expressed more than one marker, the sum total percentage of all the markers exceeds 100%. To characterise cells *in-vivo*, only the animals that had the majority of the transplant in the suprapyramidal layer were included for data analysis. Every 12^th^ section was processed for staining. To determine transplant survival, tissues sections staining with anti-GFP antibody were processed for DAB reaction. The number of GFP+ cells was counted using the optical fractionator method with the Stereo Investigator programme (MBF Bioscience). Briefly, a region of interest was drawn around the entire DG on each tissue section. This region was then divided into unbiased fractionator samples, where the optical dissector was placed. The number of GFP+ cells in each dissector was counted and the data was then used to estimate the total number of surviving cells. To determine co-localisation, tissue sections were processed for fluorescent staining. Optical sections (Z = 0.5 µm) of confocal images were acquired using a confocal laser-scanning microscope with ×40 or ×63 objective (Leica). Co-localization was determined through orthogonal sectioning by comparing signals on the same z planes to avoid false positives. The numbers of marker positive cells within the GFP+ transplant were counted (e.g. NeuN+ and GFP+ cells). The percentage of marker positive cells was calculated by the dividing the number of marker positive GFP+ cells by the total number of GFP+ cells.

### Statistics

Data are presented as mean ± SEM. Unpaired Student *t*-tests were used for between group comparisons. Probability levels p<0.05 were considered to be significant.
